# Knowledge, Attitude, and Practice of Parents Regarding Children’s Eye Care in Al-Qunfudah Governorate, Saudi Arabia

**DOI:** 10.7759/cureus.48044

**Published:** 2023-10-31

**Authors:** Safa H Alkalash, Haneen Y Alsayed, Taif k Alamshani, Bashayer A Almarhabi, Khadijah N Alsayed, Ghady M Alsayed, Raghad S Alqarni, Amirah I Alkinani, Amirah R Alsharif, Amal A Aljohani, Fuad M Alkudaysi

**Affiliations:** 1 Community Medicine and Healthcare, Umm Al-Qura University, Al-Qunfudah, SAU; 2 Family Medicine, Menoufia University, Shibin Al Kawm, EGY; 3 Medicine and Surgery, Umm Al-Qura University, Al-Qunfudah, SAU; 4 Emergency Medicine, South Al-Qunfudah General Hospital, Al-Qunfudah, SAU

**Keywords:** saudi arabia, practice, parents, knowledge, eye care, children, al-qunfudah, attitude

## Abstract

Background: Visual impairment and blindness have a long-term impact on children. Common causes include refractive error, amblyopia, and cataracts, all of which are preventable.

Objectives: This study aims to evaluate the knowledge, attitude, and practice of parents regarding children's eye care in Al-Qunfudah governorate, Saudi Arabia.

Methods: A cross-sectional study was carried out on a convenience sample of 403 parents residing in the governorate of Al-Qunfudah. Data were gathered using a validated, self-administered online questionnaire that required participants' consent and ensured data confidentiality. IBM SPSS Statistics for Windows, Version 26.0 (Released 2019; IBM Corp., Armonk, New York, United States) was used to do the statistical analysis of the data.

Results: This study included 403 participants; 41.2% of them were 36-45 years old, 75.9% were female, and 88.8% were married. Most of the participants had had their children undergo eye examinations (65%). A positive attitude about child eye care among the participants was observed in 48.9% of them, while the level of knowledge about eye care and its regular examinations was poor in 60% of the study sample. A significant positive correlation was detected between participants' attitudes and knowledge scores about the eye care of their children (r = 0.238, p-value = <0.001). Older adults (P = 0.004), those with employment (P = 0.004), and those with a history of children suffering from eye problems (P = 0.018) are associated with a positive attitude, while better knowledge is associated with the parent's age (P = 0.007) and higher education level (P = 0.047).

Conclusion: The knowledge and attitude of the parents regarding their children’s eye care were suboptimal, and the main reason for conducting eye examinations on their children was the presence of active eye disease and a symptomatic child. Positive attitudes were more prevalent among those aged 36-45 years, employed, and with a higher number of children with eye problems. Those in the age group of 36-45 who had a bachelor’s degree were associated with greater knowledge. There is an urgent need to educate parents about routine and recommended eye screening. Family physicians should conduct opportunistic eye screenings for children who attend primary healthcare facilities and provide parents with educational materials about common eye disorders and children’s eye care.

## Introduction

Blindness and visual impairment (VI) are more uncommon in youngsters than in adults. The long-term effects on children, however, are very significant [1]. According to a systematic review, amblyopia and cataracts are the two most common causes of VI in children worldwide, followed by refractive error. In addition, cataracts are the primary cause of blindness in youngsters [2]. Other causes of VI include neural generation, glaucoma, vitamin A deficiency-related corneal ulcers, and retinal disease [1]. Other typical causes of blindness include neurological conditions [3]. Numerous causes of VI and childhood blindness are defined as curable, preventative, and avoidable in developing nations [3,4].

According to estimates made by the World Health Organization (WHO) in 2017, there are roughly 19 million children under the age of 15 who have VI and 1.4 million children who have irreversible blindness worldwide. There are many disorders that cause blindness, which can be treated and prevented in half of these cases [5]. VI is a significant financial burden worldwide. For example, it is estimated that untreated myopia and presbyopia cost the world $244 billion and $25.4 billion annually, respectively [6]. In Saudi Arabia, several studies in various areas showed the frequency of VI and blindness. In Riyadh, 5% of youngsters have VI, and 2% are blind [7]. In a study in the eastern province which included 818 pediatric ophthalmology patients, blindness was seen in 22.9% of children and 71.2% had VI [8]. In a study in Arar City, 10.2% of children had VI and 0.04% were blind [9].

Early detection and treatment of eye conditions can avert vision loss and the long-term complications that follow because impairment in sight may impair early development and learning, resulting in lifelong intellectual, emotional, and social sequelae [10,11]. As a result, screening recommendations exist to direct medical professionals in identifying the most prevalent dangers to vision during the various phases of infancy and childhood [10]. The American Academy of Family Physicians and the United States Preventive Services Task Force recommend vision screening at least once in all children three to five years of age (B recommendation) [12,13]. The purpose is to detect risk factors and visual abnormalities that necessitate treatment by a family physician and to identify those patients who require a referral to an ophthalmologist skilled in examining children [12]. In Saudi Arabia, children are only required to have an eye screening test when they enter elementary school (after the age of five). Other practitioners prefer to adopt the vision screening recommendations of the American Academy of Pediatrics (AAP) and the American Academy of Pediatric Ophthalmology and Strabismus (AAPOS), which recommend an age-appropriate screening with referral criteria [14-17].

Parents' knowledge of potential visual disorders at a younger age and receiving the screening outcomes of children who failed visual screening could be essential for seeking health counseling [18]. Specifically, parents, as caregivers, play a fundamental role in seeking eye care services for their children to avoid experiencing visual disorders that may go untreated [19]. Numerous parts of the world have researched parents' knowledge of pediatric eye care. In India, parents have been seen to lack a sufficient grasp of eye diseases and their symptoms [20], and in Nigeria, parents were found to hold false beliefs about the etiology of eye diseases [19]. It has been noted that parents in Saudi Arabia have limited knowledge of refractive error, amblyopia, and eye care [14-16].

Furthermore, studies in Riyadh [21] and Madinah [22] reported that parents had little knowledge of juvenile eye problems. Despite the few Saudi studies that assessed the parents' awareness and practice regarding their children's eye care and regular examinations, none of them evaluated the risk factors of poor knowledge, attitude, and practice of the parents regarding this important issue or the correlation between parents' knowledge, attitude, and practice. Additionally, no studies have evaluated parents' awareness of children's eye care in the Al-Qunfudah governorate. Thus, the aim of this study was to assess the knowledge, attitude, and practice of parents regarding children’s eye care in Al-Qunfudah governorate, Saudi Arabia.

## Materials and methods

Study design and setting

This was a community-based cross-sectional study conducted in Al-Qunfudah governorate, Saudi Arabia, over a period of two months (August through September 2022). Al-Qunfudah governorate, a province of Makkah, was selected for the study location. It is located on the Tihamah plain on Saudi Arabia's Red Sea coast. This study targeted any adult parent who had one child or more and lived in Al-Qunfudah governorate, Saudi Arabia. The Institutional Review Board of Umm Al-Qura approved the study (HAPO-02-K-012-2022-09-1207). Consent was obtained from all participants before the questionnaire was filled out. No personal identifying data was collected from participants, and all data was coded and handled carefully to ensure its safety. The research data was stored securely on transportable media (a flash memory device and a portable external drive), and handled only by the principal investigator and authorized researchers.

Sample size calculation

Based on the total population of Al-Qunfudah governorate (300,516) and the percentage of good knowledge about eye examination among children (25.9%) from a previous Saudi study [14], taking into account a 5% margin of error and a 95% confidence interval (CI), it was determined with the help of the Raosoft sample size calculator (Raosoft Inc., Seattle, Washington, United States) that the minimum sample size should be 295. The study included 403 participants who were selected using the non-probability sampling approach, which was less expensive to implement as it saved both money and time and was the most accessible to collect data from a large parent population who accepted to participate.

Data collection

A semi-structured, pretested online questionnaire was used to collect the data, which was guaranteed to remain anonymous. The questionnaire was created by the study's researchers following a review of the relevant literature [14,15,21-23] and consultation with experts in the field who evaluated the items' clarity and relevance to the study objectives. The questionnaire was divided into four sections: (i) The first section consisted of eight items from the socio-demographic profile, such as age, education, occupation, marital status, and the number of children, (ii) The second section involved four questions about parents' practices toward children's eye care, including whether they have had eye examinations for their children, the indications, and the reasons that made them refuse to perform eye examinations, (iii) The third section, consisting of 10 items, inquired about parents' knowledge of eye problems, their understanding of refractive errors, their prevention, and complications, in addition to their knowledge of amblyopia and cataracts, and (iv) The fourth and last portion of the survey assessed the parents’ attitudes toward eye examination for their children and included six items including their perception of their child wearing eyeglasses, whether wearing eyeglasses acts as an opportunity for learning, or whether eyeglasses could deteriorate the visual acuity of their children and have a negative psychological impact on their children.

The survey was created using Google Forms (Google LLC, Mountain View, California, United States) and then pretested in a pilot study to assess whether it would be understandable by participants of various educational backgrounds and levels, as well as to establish response rates. After being shared on Al-Qunfudah Snapchat (Snap Inc., Santa Monica, California, United States) for a few days, the survey link was purposefully disabled until we had done our analysis of the early data we had collected. This pilot involved the first 30 submitted answers (representing 10% of the estimated sample size). When we evaluated the reliability of the survey, Cronbach's alpha coefficient was 0.78. The primary study results did not contain the data from the pilot trial. The required study data were gathered over a two-month period, from August to September 2022, with the distribution of the designed survey on several electronic channels for Al-Qunfudah, including WhatsApp (Meta Platforms, Inc., Menlo Park, California, United States), Twitter (X Corp., San Francisco, California, United States) and Snapchat.

To confirm that all data were obtained from Al-Qunfudah participants, the survey asked each participant about their residence, and non-residents were not included in the study's final conclusions. The response rate on this survey was 95.5%, as we received 422 responses, and after filtering them, we found 11 incomplete responses and eight responses that came from outside the Al-Qunfudah governorate. These were disregarded from the data analysis. Ultimately, 403 questionnaires were filled out in total. This high response rate suggests that there is a higher chance that the sample is representative of the target population. As a result, the validity of the survey results has increased.

Scoring system

To analyze the knowledge levels about eye problems and their screening, a score was calculated based on the respondent’s answers to the 10 knowledge-related items, which were simple-choice questions (yes or no). Incorrect answers, or if the respondent replied, “I do not know,” were given a score of 0, while correct answers were scored 1. Thus, a total knowledge score of 0-10 was calculated. The same was applied to the attitude score and the total score was 0-6. More than 75% of correct or accepted answers was considered to indicate good knowledge and a positive attitude, while 50-75% was considered to indicate fair knowledge and a neutral attitude, and those who correctly answered less than 50% were judged to have poor knowledge and a negative attitude toward eye care and regular eye examination for children [22].

Data analysis

The data were analyzed statistically using the IBM SPSS Statistics for Windows, Version 26.0 (Released 2019; IBM Corp., Armonk, New York, United States). To assess the relationship between variables, qualitative data was expressed as numbers and percentages, and the Chi-squared test (χ2) was used. Quantitative data was presented as mean and standard deviation (mean ± SD), and non-parametric variables were tested using the Kruskal-Wallis test. Correlation analysis was performed using the Spearman's test. The odds ratio (OR) was calculated at a confidence interval (CI) of 95%, and a p-value of less than 0.05 was considered statistically significant.

## Results

The total number of participants was 403, with 41.2% (n=166) of the participants in the age group of 36-45 years, 75.9% (n=306) being female, 99.9% (n=400) having a Saudi nationality, and 88.8% (n=358) being married. Of them, 63.8% (n=257) were employed, and 55.1% (n=222) had a bachelor's degree in education. The mean number of children was 3.5 ± 2.22, and the mean number of children with eye problems was 0.89 ± 1.04 (Table 1).

**Table 1:** Distribution of participants according to demographic characteristics (N= 403)

Variable	N. (%)
Age	
18-25 years	40 (9.9)
26-35 years	96 (23.8)
36-45 years	166 (41.2)
46-60 years	95 (23.6)
>60 years	6 (1.5)
Gender	
Female	306 (75.9)
Male	97 (24.1)
Nationality	
Saudi	400 (99.9)
Non-Saudi	3 (0.7)
Marital status	
Widowed	14 (3.5)
Unmarried	8 (2)
Married	358 (88.8)
Divorced	23 (5.7)
Employment status	
Student	30 (7.4)
Unemployed	116 (28.8)
Employed	257 (63.8)
Education level	
Primary School	11 (2.7)
Middle School	8 (2)
Secondary School	43 (10.7)
Bachelor's Degree	222 (55.1)
Diploma	94 (23.3)
Master's Degree	23 (5.7)
Doctorate	2 (0.5)
Number of Children	3.5 ± 2.22
Number of Children with Eye Problems	0.89 ± 1.04

Most of the participants had taken their children for an eye examination; this represented 65% (n = 262). Of these, the most common age group of their child was 6-10 years in 32.8% (n = 86). The main reason for the eye examination was the presence of eye problems (71.0%; n = 186). For those who did not take their child for an eye examination (35.0%, n = 141), the most common reason was not noticing any signs that would prompt them to take them to an eye doctor (54.2%, n = 76) (Table 2).

**Table 2:** Parents responses towards child eye examination (N= 403)

Variable	N. (%)
History of children’s eye examination	
No	141 (35)
Yes	262 (65)
The age of the child when first taken for an eye examination (N= 262)	
Less than 1 year	24 (9.1)
1-5 years	80 (30.5)
6-10 years	86 (32.8)
11-15 years	72 (27.6)
The reason for conducting eye examination (N= 262)	
Presence of eye problem	186 (71.0)
Routine examination before school	76 (29.0)
The reason for not conducting eye examination (N= 141)	
Child is too young to have an eye test	4 (2.8)
Worry about the cost of an eye test and glasses	1 (0.7)
Worry that child may be given glasses that they don’t need	3 (2.1)
No public awareness highlighting the importance of eye exams	2 (1.4)
Did not know how and/or where to arrange an appointment for an eye test	1 (0.7)
There are no specialized eye care clinics	7 (4.9)
Did not know that the child needed to be examined	47 (33.3)
Did not notice any signs that would prompt me to take them to an eye doctor	76 (54.2)

It was revealed that only 26.1% (n=105) had ever heard of refractive error from medical physicians, and 32.5% (n=131) knew that refractive error is a preventable and treatable eye condition. Of them, 35.2% (n=142) thought that refractive error could cause VI or blindness in children if left untreated. The majority, 63.5% (n=256), have heard of amblyopia (lazy eye) from their healthcare providers, and 53.8% (n=217) knew that amblyopia is a preventable and treatable eye condition. About half of the participants, 50.9% (n=205), knew that amblyopia can cause VI or blindness in children if left untreated. As for cataracts, 38.5% (n=155) had heard of them from their healthcare providers, 39.2% (n=158) knew that cataracts are a preventable and treatable eye condition, and 41.4% (n=167) knew that cataracts can cause VI or blindness in children if left untreated. Only 11.7% (n=47) of the participants knew that all children should have regular eye examinations (Table 3).

**Table 3:** Participants' knowledge about child eye care (N= 403)

Variable	No, N (%)	Do not know, N (%)	Yes, N (%)
Know about refractive error from their healthcare providers	298 (73.9)	---	105 (26.1)
Refractive error is a preventable and treatable eye problem	12 (3)	260 (64.5)	131 (32.5)
Refractive error can cause visual impairment or blindness in children when left untreated.	10 (2.5)	251 (62.3)	142 (35.2)
Know about amblyopia (lazy eye) from their healthcare providers	147 (36.5)	---	256 (63.5)
Amblyopia is a preventable and treatable eye condition	17 (4.2)	169 (41.9)	217 (53.8)
Amblyopia can cause visual impairment or blindness in children when left untreated.	13 (3.2)	185 (45.9)	205 (50.9)
Know about cataract from their healthcare providers	248 (61.5)	---	155 (38.5)
Cataracts can be prevented and treated	12 (3)	233 (57.8)	158 (39.2)
Cataracts can cause visual impairment or blindness in children when left untreated.	8 (2)	228 (56.6)	167 (41.4)
Children should undergo regular eye examination	87 (21.6)	269 (66.7)	47 (11.7)

**Figure 1:**
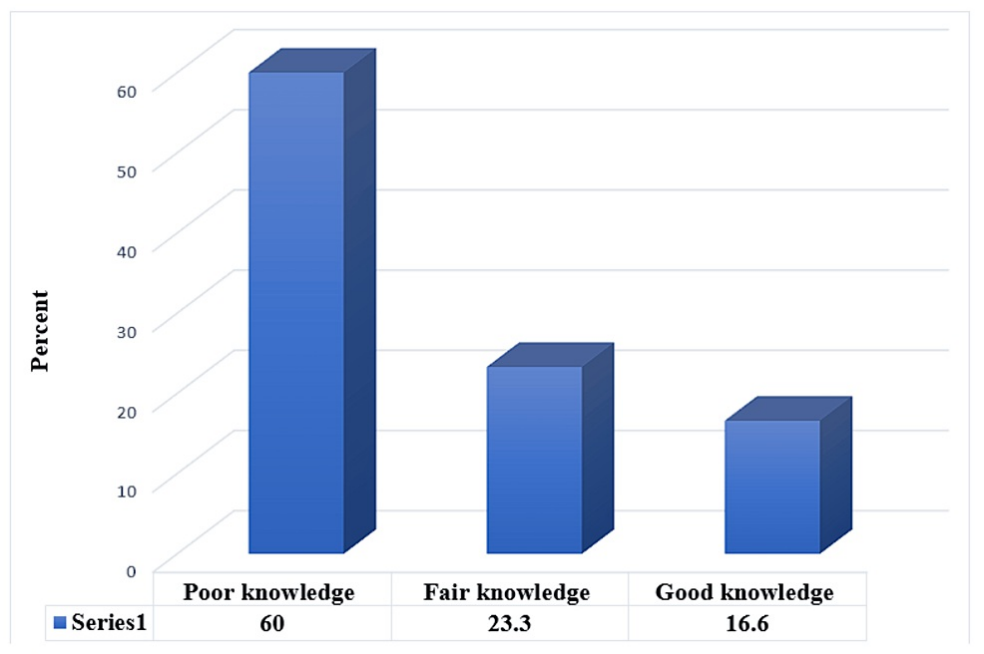
Percentage distribution of the participants according to their level of knowledge about child eye care (N= 403)

The study found that poor, fair, and good knowledge of the parents about common eye problems, their management, and the importance of child eye care were 60% (n = 242), 23.3% (n = 94), and 16.6% (n = 67), respectively (Figure 1).

Regarding the participants' attitude toward child eye care, it was found that 85.1% (n = 343) of the participants were not opposed to any of their children wearing glasses, and 81.4% (n = 328) did not think wearing eyeglasses negatively affected their child's opportunities for learning. Of them, 76.9% (n = 310) did not believe eyeglasses could reduce their child's vision, and 41.2% (n = 166) did not believe using eyeglasses has a psychological effect on children. More than half, 58.1% (n = 234), thought that the best way to prevent their children from having vision loss was through regular eye examinations (Table 4).

**Table 4:** Participants' attitude towards child eye care (N= 403)

Attitude characteristics	No, N (%)	Not sure, N (%)	Yes, N (%)
Wearing eyeglasses by the child is not a problem	343 (85.1)	30 (7.4)	30 (7.4)
Wearing eyeglasses affects the child's opportunities for learning	328 (81.4)	38 (9.4)	37 (9.2)
Eyeglasses would not reduce the child's vision	43 (10.7)	50 (12.4)	310 (76.9)
Using eyeglasses has a psychological effect on children	166 (41.2)	82 (20.3)	155 (38.5)
Wearing eyeglasses for a long time will not harm the eyes and result in early blindness	49 (12.2)	109 (27)	245 (60.8)
The best way to prevent vision loss in children is regular eye examination	89 (22.1)	80 (19.9)	234 (58.1)

**Figure 2:**
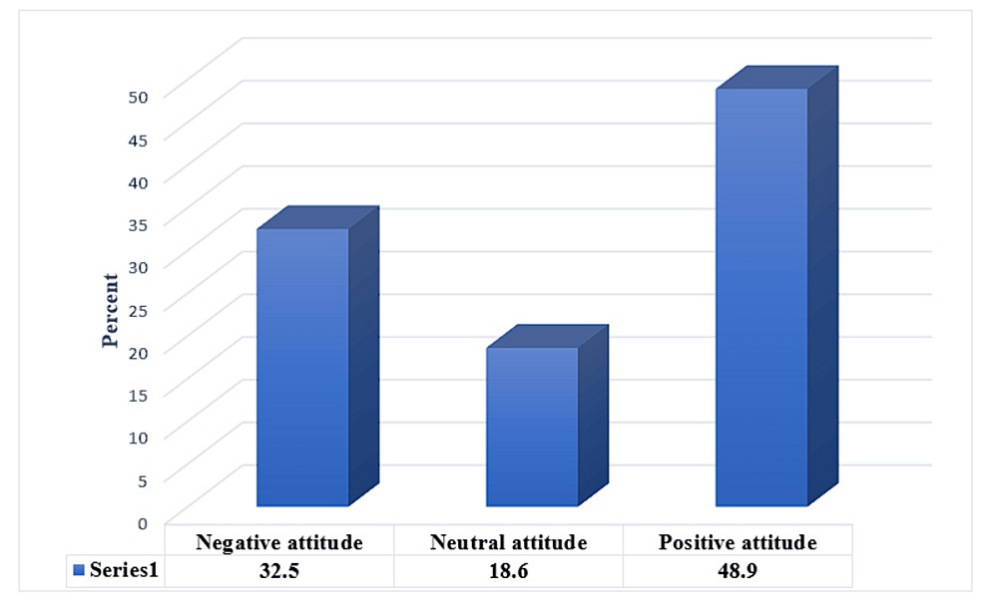
Percentage distribution of the participants according to their attitude about child eye care (N= 403)

This study revealed that 32.5% (n = 131), 18.6% (n = 75), and 48.9% (n = 197) of the parents showed negative, neutral, and positive attitudes, respectively, toward their child’s eye care, in their perceptions about regular eye examination and their acceptance of wearing eyeglasses by their children (Figure 2).

**Figure 3:**
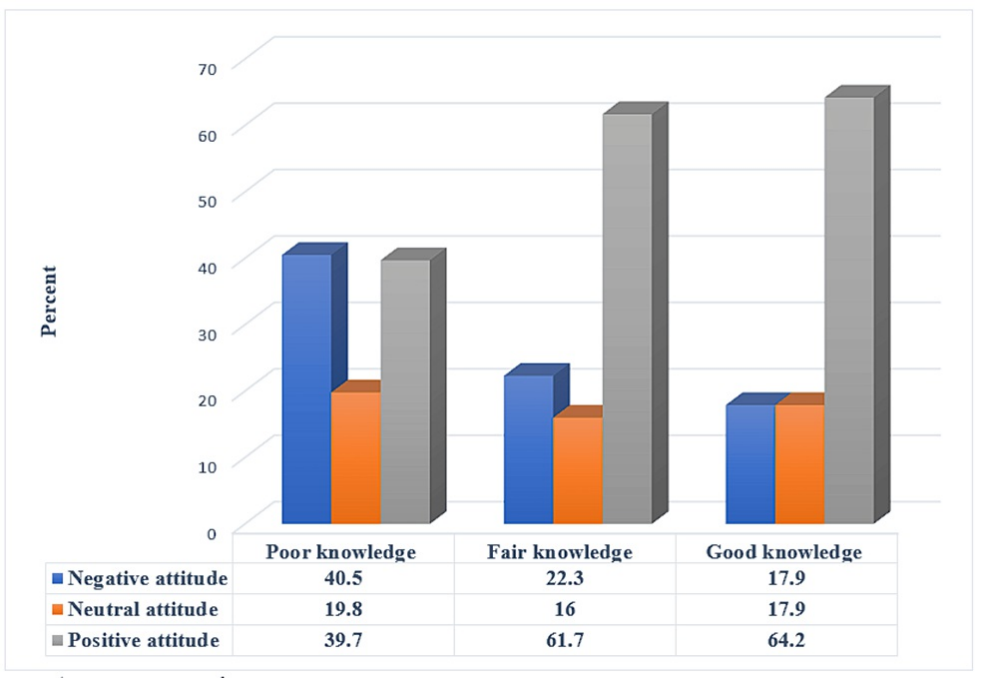
Relationship between participants' attitude and their knowledge level (N= 403) χ2 = 23.26, p-value = <0.001

There is a statistically significant relation between the parents' good knowledge level and positive attitude levels about eye care of the children (p = 0.05) (Figure 3).

**Figure 4:**
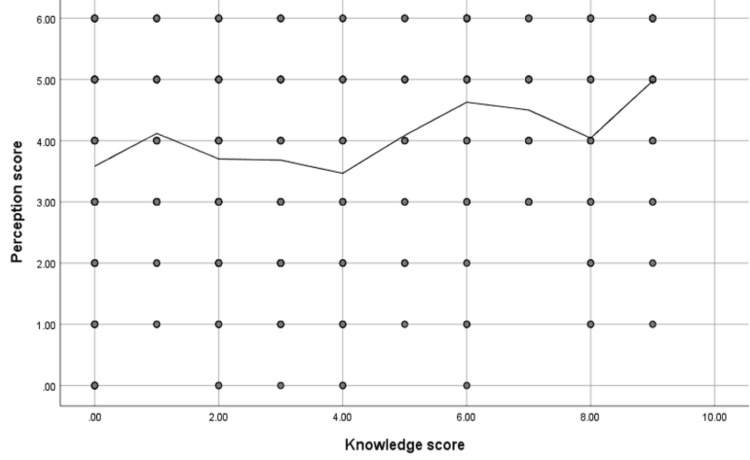
Spearman's correlation analysis between participants' attitude and knowledge scores (N= 403) r = 0.238, p-value <0.001

The study reported a significant positive correlation between participants' attitude and knowledge scores about the eye care of their children (r = 0.238, p-value = <0.001) (Figure 4).

Regarding participants' knowledge about children's eye care, the finding showed that participants who had a good knowledge level were significantly older, ranging from 36 to 45 years, and had a bachelor’s degree in education (p = 0.05) (Table 5).

**Table 5:** Relationship between participants' knowledge level about child eye care and their demographic characteristics (N= 403) ^*^ Kruskal Wallis test; p-value less than 0.05 is significant.

Variable	Knowledge level			χ2	p-value
	Poor, N (%)	Fair, N (%)	Good, N (%)		
Age					
18-25 years	25 (10.3)	8 (8.5)	7 (10.4)	21.08	0.007
26-35 years	72 (29.8)	13 (13.8)	11 (16.4)		
36-45 years	99 (40.9)	38 (40.4)	29 (43.3)		
46-60 years	42 (17.4)	33 (35.1)	20 (29.9)		
>60 years	4 (1.7)	2 (2.1)	0 (0.0)		
Gender					
Female	175 (72.3)	78 (83)	53 (79.1)	4.65	0.097
Male	67 (27.7)	16 (17)	14 (20.9)		
Nationality					
Saudi	240 (99.2)	93 (98.9)	67 (100)	0.65	0.721
Non-Saudi	2 (0.8)	1 (1.1)	0 (0.0)		
Marital status					
Widowed	7 (2.9)	3 (3.2)	4 (6)	3.61	0.728
Unmarried	6 (2.5)	1 (1.1)	1 (1.5)		
Married	215 (88.8)	83 (88.3)	60 (89.6)		
Divorced	14 (5.8)	7 (7.4)	2 (3)		
Employment status					
Student	21 (8.7)	5 (5.3)	4 (6)	5.85	0.21
Unemployed	78 (32.2)	22 (23.4)	16 (23.9)		
Employed	143 (59.1)	67 (71.3)	47 (70.1)		
Education level					
Primary School	5 (2.1)	4 (4.3)	2 (3)	21.22	0.047
Middle School	4 (1.7)	3 (3.2)	1 (1.5)		
Secondary School	33 (13.6)	5 (5.3)	5 (7.5)		
Bachelor's Degree	136 (56.2)	47 (50)	39 (58.2)		
Diploma	50 (20.7)	29 (30.9)	15 (22.4)		
Master's Degree	14 (5.8)	6 (6.4)	3 (4.5)		
Doctorate	0 (0.0)	0 (0.0)	1 (1.5)		
Number of Children	3.31 ± 2.14	3.79 ± 2.05	3.81 ± 2.67	2*	0.111
Number of Children with Eye Problems	0.81 ± 0.93	0.95 ± 1.13	1.06 ± 1.27	2*	0.582

Positive attitudes about child eye care were significantly higher among the parents in the age range of 36-45 years, who were employed, and had a higher mean number of children with eye problems (p = 0.05) (Table 6).

**Table 6:** Relationship between demographic characteristics of participants and their attitude toward eye care of their children (N= 403) ^*^ Kruskal Wallis test; p-value less than 0.05 is significant.

Variable	Attitude			χ2	p-value
	Negative, N (%)	Neutral, N (%)	Positive, N (%)		
Age					
18-25 years	19 (14.5)	4 (5.3)	17 (8.6)	22.53	0.004
26-35 years	32 (24.4)	14 (18.7)	50 (25.4)		
36-45 years	50 (38.2)	46 (61.3)	70 (35.5)		
46-60 years	27 (20.6)	10 (13.3)	58 (29.4)		
>60 years	3 (2.3)	1 (1.3)	2 (1)		
Gender					
Female	57 (76)	57 (76)	154 (78.2)	1.37	0.503
Male	36 (27.5)	18 (24)	43 (21.8)		
Nationality					
Saudi	130 (99.2)	75 (100)	195 (99)	0.75	0.684
Non-Saudi	1 (0.8)	0 (0.0)	2 (1)		
Marital status					
Widowed	5 (3.8)	3 (4)	6 (3)	2.87	0.825
Unmarried	2 (1.5)	1 (1.3)	5 (2.5)		
Married	117 (89.3)	69 (92)	172 (87.3)		
Divorced	7 (5.3)	2 (2.7)	14 (7.1)		
Employment status					
Student	15 (11.5)	3 (4)	12 (6.1)	15.39	0.004
Unemployed	48 (36.6)	14 (18.7)	54 (27.4)		
Employed	68 (51.9)	58 (77.3)	131 (66.5)		
Education level					
Primary School	6 (4.6)	0 (0.0)	5 (2.5)	13.77	0.315
Middle School	4 (3.1)	1 (1.3)	3 (1.5)		
Secondary School	20 (15.3)	5 (6.7)	18 (9.1)		
Bachelor's Degree	64 (48.9)	45 (60)	113 (57.4)		
Diploma	28 (21.4)	19 (25.3)	47 (23.9)		
Master's Degree	9 (6.9)	5 (6.7)	9 (4.6)		
Doctorate	0 (0.0)	0 (0.0)	2 (1)		
Number of children	3.15 ± 2.13	3.61 ± 2.01	3.69 ± 2.34	2*	0.076
Number of children with eye problems	0.66 ± 0.83	0.89 ± 0.92	1.03 ± 1.18	2*	0.018

A multivariate logistic regression analysis was done to assess the factors (independent predictors) of good knowledge levels among the participants. It was found that none of the studied variables were found to be risk factors (independent predictors) of good knowledge level (Table 7).

**Table 7:** Multivariate logistic regression analysis of factors associated with good knowledge level among participants.

Variable	Knowledge level			
	B	Wald	p-value	OR (95%CI)
Age	0.03	0.03	0.846	0.96 (0.66-1.39)
Gender	0.01	0.37	0.591	0.09 (0.15-1.07)
Nationality	0.06	0.71	0.391	0.12 (0.16-1.05)
Marital status	0.42	2.41	0.12	0.65 (0.38-1.11)
Employment status	0.24	0.69	0.404	1.27 (0.71-2.26)
Education level	0.04	0.06	0.796	10.04 (0.76-1.4)
Number of children	0.04	0.36	0.549	1.04 (0.9-1.19)
Number of children with eye problems	0.14	1.21	0.271	1.15 (0.89-1.49)

The multivariate logistic regression analysis predicted that having a higher mean number of children with eye problems was a factor in predicting a positive attitude about child eye care (Table 8).

**Table 8:** Multivariate logistic regression analysis of factors associated with positive attitude among studied participants. p-value less than 0.05 is significant.

Variable	Attitude			
	B	Wald	p-value	OR (95%CI)
Age	0.01	0.01	0.891	0.98 (0.74-1.28)
Gender	0.2	0.31	0.541	0.02 (0.13-1.01)
Nationality	0.76	0.37	0.543	2.13 (0.18-4.74)
Marital status	0.12	0.33	0.565	1.13 (0.73-1.75)
Employment status	0.14	0.74	0.492	1.15 (0.76-1.74)
Education level	0.04	0.13	0.781	1.04 (0.83-1.3)
Number of children	0.02	0.17	0.679	1.02 (0.92-1.13)
Number of children with eye problems	0.24	5.05	0.024	1.28 (1.03-1.58)

## Discussion

One requirement for engaging in health-seeking activity is knowledge of diseases and associated symptoms [24]. This is crucial since parents are the children's primary caregivers and have a significant role in their behavior while seeking eye care. It is also crucial because early detection and intervention for VI can have a positive impact on a child's future. The aim of this study was to assess the parents' knowledge, attitude, and practice of children’s eye care in Al-Qunfudah governorate, Saudi Arabia.

The present study revealed that most of the participants had taken their child for an eye examination, 65% (n = 262), while the most common reason for parents who had not taken their child for an eye examination was not noticing any signs that would prompt them to take them to an eye doctor (54.2%, n = 76 out of 141). One Saudi study done in Al-Madinah found that over half of the participants, 58.6% (n = 325), reported that they had visited ophthalmology clinics for the examination of their children. Moreover, the most common reason for parents who had not taken their child for an eye examination was the absence of signs that would prompt them to seek medical advice [22]. Another study done in Aseer revealed that 48.5% (n = 436) of the respondent parents accompanied their children to eye examinations. Furthermore, for parents who never take their children to an eye examination, the most common reason is that they assert that their children can see well [25]. Therefore, it is very important to design and implement health education campaigns to discuss the advantages of regular eye examinations and early detection of any VI among children and warn them about the probable complications resulting from discovering eye diseases in their late stages.

According to the current study, 63.5% (n = 256), 38.5% (n = 155), and 26.1% (n = 105) of parents have, respectively, heard about amblyopia, cataracts, and refractive error. Similar to the current study, 26.2% of participating parents in Riyadh City were aware that children can have refractive defects [21]. Furthermore, 49.7% and 58.5% of study participants were knowledgeable about amblyopia in Jeddah [26] and Madinah [22], respectively. These percentages were lower than in the present study; the variations in the results may be attributable to differences in the characteristics of the study subjects.

Some eye conditions are preventable, while others are not. This necessitates a different approach to interventions to decrease the risk of blindness and VI and to reduce the impact of these conditions on patients. These interventions include health promotion, prevention, treatment, and rehabilitation, as the WHO reported. Health promotion focuses on interventions that affect people's behaviors and attitudes to achieve better eye care habits [27]. In this study, only 32.5% (n = 131) and 39.2% (n = 139) of the parents recognized that refractive errors and cataracts can be managed, while about 53% of them knew that amblyopia is a preventable and treatable eye disease. Contrary to this study finding, a previous Saudi study reported that 74.0% of their study participants cited eyeglasses as a suitable treatment for refractive errors among children [23].

The level of good knowledge in this study was 16.6% (n = 67), compared to poor knowledge at 60% (n = 242). Poor knowledge was also high in previous studies done in Saudi Arabia, such as a study in Jeddah that found low awareness levels about amblyopia among pediatric and ophthalmology clinic attendees [26]. In Riyadh, poor knowledge of eye health and care among participants was noted by 91.9%, and only 26.2% knew about refractive errors [22], while in Madinah, the percentage of poor knowledge was 78.2% [22]. Despite the fact that the level of knowledge was relatively higher than the previous Saudi studies, the Saudi parents' knowledge about their child’s eye care is still poor, and there is an urgent need to educate parents about this important health-related issue.

The current study revealed that 85.1% (n = 343) of the participants had no problem when any of their children required wearing glasses. This positive attitude rate is higher than previous relevant studies done in Saudi Arabia [22,28]. This finding is promising, as the stigma of wearing eyeglasses is a common source of non-compliance to correct errors of refraction among children.

This study found an important positive correlation between participants' attitudes toward their children's eye examinations and knowledge scores, which is consistent with the findings of a South African study [29]. This positive correlation is a logical one because when someone has enough information about a certain health issue, his or her knowledge may guide him or her to perceive it adequately.

However, participants’ levels of negative, neutral, and positive attitudes about child eye care were 32.5% (n = 131), 18.6% (n = 75), and 48.9% (n = 197), respectively, in this study. This finding is against that obtained by Surrati et al. [20], where the parents' attitudes regarding children wearing eyeglasses and undergoing ophthalmic surgery when needed were positive in 76.9% and 85.4% of parents, respectively. It is possible that participants had a positive attitude toward child eye care based on their personal experiences or observations rather than objective knowledge. Despite a positive attitude towards eyewear and eye health, a significant number of parents don’t opt for regular eye check-ups for their children due to a lack of awareness. This highlights the urgency of health education campaigns about this important health issue.

Parents with bachelor's degrees and those aged 36-45 years possessed better knowledge than the others, which was similar to the results of another study [25]. Expectedly, a higher education level and a younger age are usually associated with more opportunities to learn and read about health issues. Positive attitudes toward children's eye examinations were higher among employed parents in the age group of 36-45 years and those with a history of more children with eye problems. Being an employed adult increases the likelihood of having the opportunity to pick up more knowledge and share experiences with others to better understand the importance of regular eye examinations to maintain healthy vision. Therefore, we can say that the parents' socio-economic factors might play a role in their knowledge and attitude towards their children's eye examination, and this finding is supported by the conclusion of a systematic review that expressed the role of family socioeconomic status on the utilization of pediatrics' eye examination [30].

Limitations

There are some limitations to this study. First, we caution other researchers who look for public knowledge about any health-related issues to assess its source. The study did not examine the sources of information about eye conditions among the study participants, which may have an impact on their knowledge accuracy and could have been helpful for developing educational campaigns. Second, the online nature of the study may have unintentionally skewered the sample in favor of a group with superior internet access or technological competence, in addition to non-response bias. For this reason, we suggest that future daughter studies employ in-person interviews with parents. As a result, parents who are illiterate or technologically incapable can take part in the study. Third, the use of non-probability samples could limit how broadly the study's conclusions can be applied. We advocate undertaking additional research on this subject using random sampling techniques. Despite the drawbacks described above, this work encourages further studies with different research designs to clarify different perspectives on eye care and regular eye examinations among the young population.

## Conclusions

The knowledge and attitude of parents about child eye care were clearly suboptimal in the Al-Qunfudah governorate. It was seen that parents request eye examinations for their children in light of a new complaint, not to screen regularly for non-symptomatic eye disorders that will affect the child's vision. Older adults, those with employment, and those with a history of children suffering from eye problems are associated with a positive attitude, while better knowledge is associated with the parent's age and higher education level. Parents should be targeted for health education to enhance and encourage eye care awareness for their children since numerous childhood blindness and VI causes are preventable and could be avoided. We recommend health awareness programs and campaigns in schools, hospitals, and communities, in addition to utilizing social media to increase public awareness, including parents, about children's eye care, highlighting the benefits of routine eye exams, early identification of any VI in children, and alerting them about the potential consequences of finding eye illnesses in their latter stages. Furthermore, regular eye examinations of the children in daycare centers and schools by school healthcare providers may limit the problem of non-adherence by the parents to regular eye examinations for their children. Primary care physicians can perform ophthalmologic screening for any children who come to the family health center for other health care services, in addition to educating parents about the importance of regular eye examinations and providing them with educational materials.
